# Host DNA integrity within blood meals of hematophagous larval gnathiid isopods (Crustacea, Isopoda, Gnathiidae)

**DOI:** 10.1186/s13071-019-3567-8

**Published:** 2019-06-24

**Authors:** Gina C. Hendrick, Maureen C. Dolan, Tanja McKay, Paul C. Sikkel

**Affiliations:** 1 Department of Biological Sciences, Arkansas State University, State University, AR 72467 USA; 2 Arkansas Biosciences Institute, 504 University Loop, Jonesboro, AR 72401 USA

**Keywords:** Marine parasite, DNA degradation, Blood meal analysis, *cox*1 barcoding

## Abstract

**Background:**

Juvenile gnathiid isopods are common ectoparasites of marine fishes. Each of the three juvenile stages briefly attach to a host to obtain a blood meal but spend most of their time living in the substrate, thus making it difficult to determine patterns of host exploitation. Sequencing of host blood meals from wild-caught specimens is a promising tool to determine host identity. Although established protocols for this approach exist, certain challenges must be overcome when samples are subjected to typical field conditions that may contribute to DNA degradation. The goal of this study was to address a key methodological issue associated with molecular-based host identification from free-living, blood-engorged gnathiid isopods—the degradation of host DNA within blood meals. Here we have assessed the length of time host DNA within gnathiid blood meals can remain viable for positive host identification.

**Methods:**

Juvenile gnathiids were allowed to feed on fish of known species and subsets were preserved at 4-h intervals over 24 h and then every 24 h up to 5 days post-feeding. Host DNA extracted from gnathiid blood meals was sequenced to validate the integrity of host DNA at each time interval. DNA was also extracted from blood meals of wild-fed gnathiids for comparison. Attempts were also made to extract host DNA from metamorphosed juveniles.

**Results:**

Using a *cox*1 universal fish primer set, known fish host DNA sequences were successfully identified for nearly 100% of third-stage juvenile gnathiid blood meals, digested for up to 5 days post-feeding. For second-stage juveniles, host identification was 100% successful when gnathiids were preserved within 24 h of collection. Fish hosts were positively identified for 69% of sequences from wild-fed gnathiid isopods. Of the 31% of sequences not receiving a ≥ 98 % match to a sequence in GenBank, 25 sequences were of possible invertebrate origin.

**Conclusions:**

To our knowledge, this is the first study to examine the degradation rate of gnathiid isopod blood meals. Determining the rate at which gnathiids digest their blood meal is an important step in ensuring the successful host identification by DNA-based methods in large field studies.

**Electronic supplementary material:**

The online version of this article (10.1186/s13071-019-3567-8) contains supplementary material, which is available to authorized users.

## Background

Parasites are highly diverse organisms, found within every phylum and comprise approximately half of all living organisms [1–3]. The myriad of life history strategies of parasitic organisms (e.g. host specialists *vs* host generalists; endoparasites *vs* ectoparasites) complicates the assessment of host-exploitation patterns in ecological communities. The act of parasitism influences individual hosts by affecting growth, behavior, and reproductive success [4] and therefore impacts population and community dynamics [3, 5, 6]. Thus, understanding patterns of exploitation of hosts by parasites is essential for understanding host populations and community processes.

Among ectoparasites of vertebrates, hematophagous, or blood-feeding, arthropods are perhaps the best-known and most thoroughly studied. Feeding on blood and other bodily fluids of their hosts, these organisms can impact hosts by removal of vital fluids and facilitating infection [7]. Many are also vectors for pathogenic blood parasites, viruses, and bacteria. The most notorious of these are certain species of mosquitoes and ixodid ticks [8–10]. For those that feed on multiple host species, it is particularly difficult to assess patterns of host exploitation, since one cannot rely solely on collection of parasites from hosts, as this could lead to a sampling bias based on the ability to capture and process hosts.

Gnathiid isopods are among the most common ectoparasites of marine fishes, found globally and at various depths [11–14], and thus likely exceed the biomass of ticks and mosquitoes combined. Gnathiids have been most extensively studied on coral reefs where they infect a wide range of coral reef fishes [15–17], are the primary food of cleaner fishes [18–21], and are known to be a driving factor in cleaning symbioses [21–24].

**Fig. 1:**
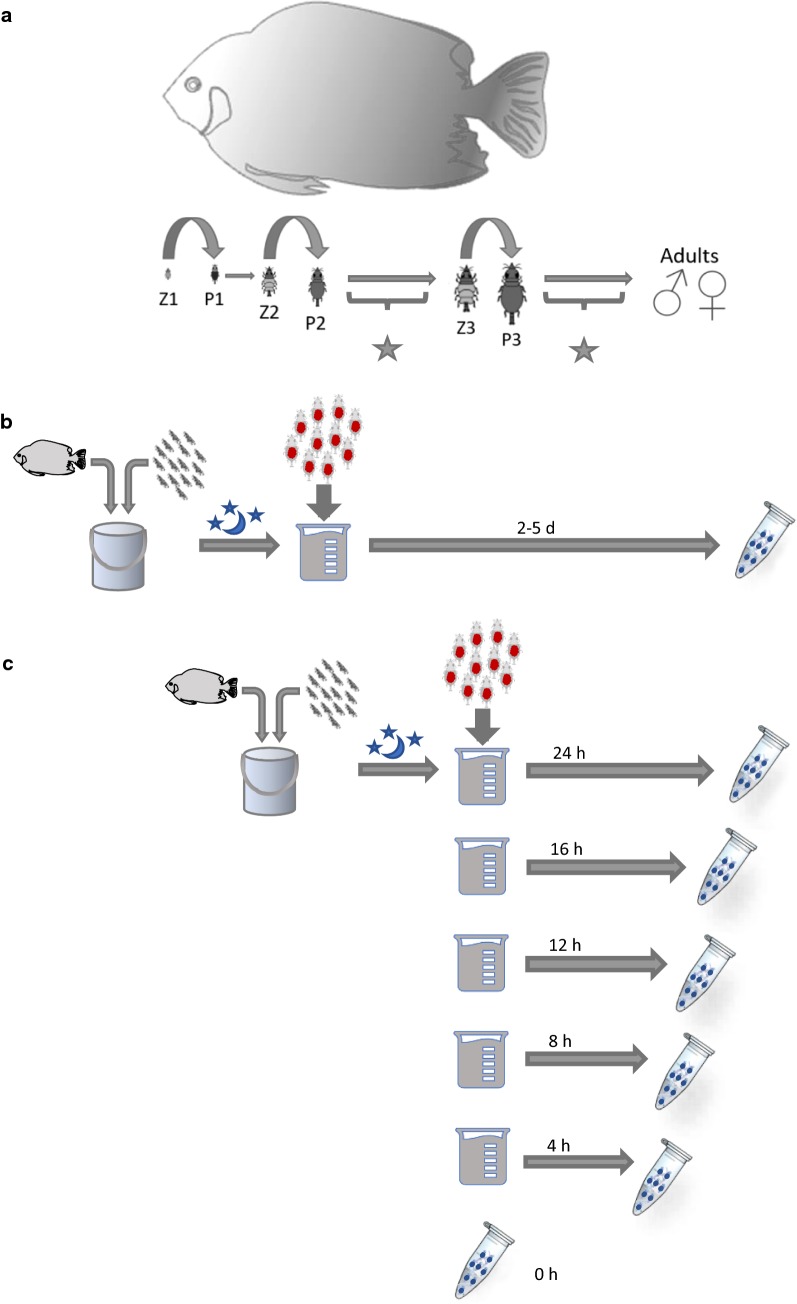
Life-cycle of gnathiid isopods and a general workflow for establishing host DNA integrity over time from gnathiid blood meal. **a** The three stages of unfed juvenile zuphea are identified as Z1, Z2 and Z3. Upon feeding, these zuphea develop into first-stage (P1), second-stage (P2), and third-stage (P3) fed pranizae, respectively. The star indicates the points within the gnathiid’s life-cycle where we assessed host DNA integrity within blood meals. **b** Fifty gnathiids of each stage were placed into one of three containers, each filled with approximately 5 l of seawater. Unfed gnathiids were allowed to feed on a fish overnight, under controlled conditions. The following morning, the fish were removed and the fed gnathiids (pranizae) were transferred to 50 ml beakers filled with seawater. Pranizae were allowed to digest their blood meals up to 5 days post-feeding. All subsets were preserved in 100% molecular grade ethanol, and at **c** 0, 4, 8, 12, 16, and 24 h post-feeding, gnathiids were preserved in real time

Gnathiids are known to feed on the blood of fishes during each of three juvenile stages [11, 12], but do not feed as adults. At each juvenile stage an unfed gnathiid, or zuphea (Z1–Z3), attaches to a single host to feed until engorged. This fed gnathiid, or praniza (P1–P3), digests the blood meal and molts, returning to the unfed zuphea form after the first and second feedings, and to adult after the third feeding (Fig. 1a).

Given that feeding only requires minutes to hours, and molting requires multiple days, gnathiids spend most of their life-cycle in the substrate, not attached to their host (the only exception to this may be species that feed on sharks, which may remain attached to hosts for long periods). Thus, like some terrestrial blood-feeding arthropods, the term “micropredator” may also be suitable [10, 25–27]. During their free-living stages, including fed (praniza) stages, gnathiids can be easily collected using lighted plankton traps [28].

DNA-based taxonomy and identification, a powerful tool incorporated into many current biomonitoring studies, has resulted in the discovery of new faunas [29]. Using DNA barcoding for species identification is becoming more accessible, reliable, and more widely adopted, supplanting traditional morphology-based species identification [30]. DNA barcoding typically targets haplotypic (maternally inherited) mitochondrial genes, such as cytochrome *c* oxidase subunit 1 (*cox*1, COI or MT-CO1), as these genes are conserved across animal taxa and a vast number of reference sequences are readily available in public databases [31]. The success in species identification using *cox*1 is largely due to a relatively fast rate of evolution of this gene, allowing for a more robust phylogenetic signal than any other mitochondrial gene [32].

The majority of studies related to interactions between blood-feeding arthropods and their hosts have focused on terrestrial species. Methodologies for DNA barcoding of hematophagous arthropod blood meals to identify hosts have been reported [31, 33, 34]. Specifically, the mitochondrial target gene *cox*1 has been successfully used to identify hosts of a variety of blood-feeding arthropods, such as ticks, mosquitoes, and biting midges [33, 35]. In aquatic systems, the use of DNA barcoding to identify fish species is well established for purposes of identifying fishes caught for human consumption [36, 37]. DNA mini-barcodes of the *cox*1 gene have been used to reproducibly identify fish species in heavily processed food products [36]. Furthermore, *cox*1 DNA sequences for a wide variety of marine fishes are publicly available [38] and provide a valuable reference database.

A particular challenge that must be properly managed when analyzing parasite blood meal samples subject to field conditions is DNA degradation. Efforts to limit or mitigate the DNA degradation process is paramount to the successful amplification of host DNA and the accurate identification of the blood meal source [31, 39]. Therefore, maintaining DNA integrity in the blood meal of ectoparasites will likely be a product of environmental conditions during field collection, proper preservation, and the digestion rate of the specific parasite.

Understanding how long after feeding a blood meal will remain viable for host identification is crucial to efficiently identifying the hosts of field-collected, blood-feeding arthropods. Determining a window of sample viability limits unnecessary sample processing costs. In this study, we assessed the integrity of host DNA within blood meals of the common Caribbean gnathiid, *Gnathia marleyi*, at multiple time intervals post-ingestion and established methods that enable reproducible host identification. *Gnathia marleyi* is thus far the only gnathiid species at our study sites and known to feed on at least 20 different host fishes [17, 40]. This information will be key in establishing a standard operating procedure for the collection, processing, and accurate host species identification of gnathiids, ultimately to be incorporated into a robust biomonitoring approach for assessing coral reef health.

## Methods

### Study species and field collection

Gnathiids were collected between May and August of 2016 and 2017. All gnathiids were collected using lighted plankton traps, following the protocol established by Artim and Sikkel [28]. Light traps were deployed before dusk and allowed to remain on the reef until retrieval at dawn the following morning. The contents of each trap were filtered and sorted under a stereomicroscope to separate living juvenile gnathiid isopods from the collected plankton.

Two subsets of gnathiids were collected: (i) gnathiids used to determine known host DNA viability within digested blood meals over time were collected in Brewer’s Bay, St. Thomas, United States Virgin Islands (USVI) (18°20ʹ25.3ʺN, 64°58ʹ36.8ʺW). These gnathiids were preserved in 100% molecular grade ethanol at specific time points specified below; (ii) gnathiids used to validate molecular methods for processing wild-fed gnathiids with unknown hosts were collected off the coast of La Parguera, Puerto Rico (17°57ʹ18.1ʺN, 67°03ʹ08.1ʺW). The latter were preserved in 100% molecular grade ethanol immediately upon sorting of plankton samples.

### Blood meal host DNA viability experimental design

Unfed juvenile gnathiids (zuphea), retrieved from the lighted plankton traps in Brewer’s Bay, St. Thomas and used to assess host DNA integrity in gnathiid blood meals, were kept in small containers filled with seawater for two days to assure that the gnathiids were starved. Fifty gnathiids of each stage (Z1–Z3) were placed into designated containers, each filled with approximately 5 l of seawater. At dusk on the third day, each container of unfed gnathiids were allowed to feed overnight on a barred hamlet (*Hypoplectrus puella*) under controlled conditions (Fig. 1b, c). Water changes were performed daily.

To assess gnathiid host DNA integrity in blood meals between day 2 and day 5 post-feeding (Fig. 1b), each time point used newly starved gnathiids and a new host fish. Following an overnight feeding, gnathiids were removed from the containers, separated based on juvenile stage, and allowed to continue digesting their blood meal for designated post-feeding time. To asses host DNA integrity in blood meals over the first 24-h post-feeding period, fed gnathiids were collected and sorted into 6 subsets of 25 individuals, for both second- and third-stage juveniles. These subsets were preserved in 100% molecular grade ethanol, at 0, 4, 8, 12, 16, and 24 h post-feeding, in real time (Fig. 1c). The 0 h time point was set at 08:00 h. Gnathiids which had metamorphosed into adult males were also preserved and analyzed separately from the individuals still in their juvenile form.

All samples, preserved in ethanol, were maintained at 24 °C for ≤ 24 h prior to being placed on ice for shipment to Arkansas State University, Jonesboro, USA, and subsequently stored at − 20 °C until processing. All specimens were processed individually and at no time were samples pooled. First-stage pranizae were not used in this study due to their high mortality rate during handling, feeding, and filtering.

### Blood meal DNA extraction, *cox*1 amplification and sequencing

DNA extraction, amplification, and sequencing methods were the same for both gnathiids used to identify unknown hosts (*n *= 100) and gnathiids used to assess host DNA integrity within blood meals (*n *= 252). DNA was extracted from whole, fed gnathiids using the PureLink® Genomic DNA extraction kit (Invitrogen, Carlsbad, CA), in accordance with the manufacturers ‘Mammalian Tissue and Mouse/Rat Tail Lysate’ protocol. Briefly, specimens were removed from ethanol, placed on a Kimwipe to wick organic solvent from the specimen, immediately transferred to a 1.5 ml microfuge tube with extraction solution and homogenized using a disposable Dounce homogenizer. Purified DNA was eluted using dH_2_O and was quantified using a NanoDrop ND-1000 UV/Vis spectrophotometer (Thermo Fisher Scientific, Wilmington, DE, USA). DNA quality and quantity of select samples were further confirmed by agarose gel electrophoresis (1.5% agarose, 1× TBE) using a DNA low mass ladder (Invitrogen, Cat no. 10068-013). Select DNA samples were visualized using ethidium bromide staining.

For all second- and third-stage pranizae evaluated, 50 ng of purified template DNA was used for each PCR amplification. Purified DNA was concentrated as needed using the ThermoSavant ISS110 SpeedVac® System (Thermo Fisher Scientific, Wilmington, DE, USA). PCR reactions were carried out using a Veriti 96 Well Thermal Cycler (Applied Biosystem, Foster City, CA, USA). Thermocycling conditions included an initial denaturation step of 94 °C for 2 min, followed by 30 cycles of 96 °C for 20 s, 55 °C for 20 s, and 72 °C for 45 s, with a final extension step of 72 °C for 7 min. Optimized *cox*1 diagnostic primers based on those listed in FishBOL (5′-TCA ACY AAT CAY AAA GAT ATY GGC AC-3′; 5′-ACT TCY GGG TGR CCR AAR AAT CA-3′) were provided by Applied Food Technologies (AFT) (Alachua, FL, USA) [37]. PCR reactions (20 µl) were carried out using 1.25 units GoTaq Hot Start Polymerase, 1× buffer with 1.5 mM MgCl_2_ (Promega, Madison, WI, USA) and 0.2 mM dNTP Mix (Thermo Fisher Scientific). PCR products were visualized on a 1.5% agarose 1× TBE (89 mM Tris, 89 mM boric acid, 2 mM EDTA, pH 8.6) gel stained with ethidium bromide to confirm target *cox*1 amplicons of expected size (540–600 bp).

ExoSAP-IT (Applied Biosystems, Foster City, CA, USA) was used to process *cox*1 PCR products prior to Sanger sequencing of each sample using the forward and reverse PCR primers indicated above (University of Chicago Comprehensive Cancer Center, DNA Sequencing & Genotyping Facility). DNA from the tissue of a fish of known species was used as a positive control for PCR amplification and DNA sequence identity. For samples that did not produce a definitive DNA sequence and chromatogram to enable successful host identification, a second PCR was performed using increased purified template DNA (100 ng). For samples whereby sufficient purified DNA template was not available to allow for this second PCR, 10 µl of the first PCR reaction provided the template source for reamplification (Table 1).

**Table 1:** Successful PCR and DNA sequencing of host DNA from gnathiid blood meals

Post-feeding (h)	Stage 2 pranizae				Stage 3 pranizae			
	1st PCR^a^	*N*	2nd PCR^b^	*N*_2_	1st PCR^a^	*N*	2nd PCR^b^	*N*_2_
0	100	10	–	–	94.7	19	100	1
4	91.7	12	100	1	100	22	–	–
8	100	13	–	–	95.8	24	95.8	1
12	94.1	17	100	1	90.5	21	100	2
16	90.5	21	100	2	86.4	22	100	3
24	100	14	–	–	89.5	19	100	2
48	15.4	13	76.9	11	100	15	–	–
72	nd	nd	nd		87.5	24	100	3
96	nd	nd	nd		84.6	13	100	2
120	nd	nd	nd		55	20	100	9

*Notes*: Percent successful identification of fish host species *Hypoplectrus puella*, using 50 ng of DNA template extracted from gnathiid blood meals in 1st PCR, at each preservation time interval (post-feeding. in h) for second- and third-stage pranizae. Percent successful identification of host species after increasing DNA template or reamplification of PCR products (2nd PCR), performed only on samples that did not amplify using 50 ng template DNA

^a^The percent successful host identification after the first PCR

^b^The percent successful host identification after the second PCR

*Abbreviations*: *N*, the total number of samples at each time point; *N*_*2*_, number of samples requiring a reamplification of PCR amplicon or increase in template DNA concentration; nd, not determined

### Host identification

Geneious R10 (Biomatters Limited, Auckland, New Zealand) software was used to generate a consensus sequence of the forward and reverse *cox*1 sequence for each gnathiid blood meal sample analyzed. This consensus sequence was entered into the Basic Local Alignment Search Tool (BLAST) on the National Center for Biotechnology Information (NCBI) website, as well as into the Barcode of Life Data Systems (BOLD) website to determine the host species identity. A ≥ 98 % match to a reference sequence using BLAST was considered a positive species identification. This was performed for gnathiids used to assess host DNA integrity within blood meals (*n *= 252) and to identify unknown hosts (*n *= 37). The forward *cox*1 sequence was used to determine the host identity of the remaining wild-fed gnathiids (*n *= 63).

## Results

### Host identification success from digested blood meals: second-stage pranizae

The host fish species, *Hypoplectrus puella*, was successfully identified from blood meals of second-stage pranizae allowed to digest for up through 48 h post-feeding (Table 1). Second-stage pranizae preserved at 0 h post-feeding (08:00 h collection time following overnight exposure to known host fish), resulted in 100% successful host identification. For each successive 4-h collection point, the success rates of host DNA species identification was greater than 90%. At 48 h, the host species identification success rate for the second-stage pranizae, dropped to 15.4% and by 72 h post-feeding, host DNA was undetectable.

### Host identification success from digested blood meals: third-stage pranizae

The host fish species, *Hypoplectrus puella*, was successfully identified from blood meals that had been digested by third-stage pranizae for up to 120 h post-feeding (Table 1). The third-stage pranizae preserved immediately upon removal from host had a 94.7% host identification, and remained ≥ 86.4 % over the first 48 h assessed. DNA extracted from gnathiid digested blood meals 72 h, 96 h, and 120 h post-feeding showed an expected progressive decrease in host identification success rates of 87.5%, 84.6%, and 55.0%, respectively. Seven third-stage pranizae metamorphosed into adult males during these trials, and the DNA within their blood meals was successfully amplified for host identification.

### Impact of PCR template DNA concentration on host identification success

Interestingly, significant gains in host identification success could be attained with select samples when increased amounts of template DNA were used for *cox*1 PCR. At the 48-h time interval, successful host identification of second-stage pranizae rose significantly from 15.4% to 76.9%. The third-stage pranizae analyzed under these reaction conditions resulted in nearly 100% successful host identification in samples up through 5 days post-feeding. For all time points assessed up to 24 h for both second and third-stage pranizae, doubling the DNA template used for PCR and reamplification of PCR amplicons, notably achieved 100% successful host identification (Table 1).

### Success of host identification from wild-fed gnathiids

Of the 100 gnathiids (stage 2–3) with unknown hosts analyzed, a BLAST search resulted in a positive host identification for 69% of samples. All positively identified hosts were fishes, representing ten families (see Additional file 1: Table S1). The remaining 31.0% of samples did not receive a ≥ 98 % match to a sequence in GenBank. Of these samples, 25 samples loosely matched to an invertebrate species.

In order to confirm the diagnostic primers did not detect gnathiid DNA, we attempted to amplify purified DNA from 18 third-stage unfed zuphea. These 18 gnathiids were previously fed at stage Z2 using a fish of known species (*Hypoplectrus puella*) and allowed to digest their blood meal for 5–6 days while metamorphosing into stage Z3. DNA extracted from these samples was used to test for PCR amplification of gnathiid DNA using *cox*1 universal fish primers. Fifteen of these gnathiids did not produce a PCR amplicon. DNA was amplified from only three of the zuphea, with sequences matching to the known host fish *Hypoplectrus puella.*

## Discussion

This study provides a systematic approach to understanding the stability of DNA in blood meals of *Gnathia marleyi* essential for developing a reliable and reproducible molecular diagnostic for gnathiid host identification. As first-stage pranizae are difficult to collect in the field, and blood meals are small, we focused on second and third-stage pranizae. While host identity can be determined up to 120 h post-feeding from third-stage juvenile blood meals and adult males, blood meals sourced from second-stage pranizae were most reliable as template when preserved during the first 24 h of gnathiid collection. This shorter window for preserving second-stage pranizae could be due to the notable differences in blood meal volume recovered at each of these juvenile stages. For the species used in this study, the final and largest blood meal (P3) in a gnathiid’s life is approximately 1 µl, whereas second-stage pranizae tend to obtain blood meals of less than 0.3 µl [27]. Another factor that may contribute to the shorter preservation window of second-stage pranizae is possible differences in gut chemistry among juvenile gnathiid stages. However, further studies are needed as proteolytic activity has only been measured for the gut of third-stage juvenile gnathiids (*Paragnathia formica*) [41].

While molecular-based host identification of second-stage pranizae is best determined from specimens collected within 24 h of trap retrieval, accurate host identification greater than 75% can still be attained from samples collected up through 48 h post-feeding. This degree of host DNA stability is surprising given that in other blood-feeding arthropods, like mosquitoes, host identification reduces to less than half within 30 h post-feeding, using similar methodologies [31, 39]. Considering collection logistics in the field and the need to collect thousands of specimens for ecologically-relevant studies, using 24 h as a cut-off for specimen preservation is a workable timeframe. It should be noted that the methods developed herein were designed to identify hosts of a Caribbean gnathiid isopod. While these methods are applicable to other gnathiid species, the retrieval times and metabolic (and hence host DNA digestion) rates may vary for specific gnathiid species. Gnathiids from polar regions, for example, require two years to complete their life-cycle [42].

Processing logistics for field collection of gnathiid specimens is an important consideration. Due to some restrictions at certain field sites, traps must be set at dusk and allowed to remain on the reef overnight and retrieved the following morning. The 0 h time point was thus set at 08:00 h to simulate the anticipated trap collection and processing time during field collection of a gnathiid, having detached from its host and immediately entered a light trap prior to its collection. As the gnathiid species used in this study (*G. marleyi*) molt to the next Z-stage (in the case of P1 and P2 larvae), or to an adult (in the case of P3 stage) in 5–6 days, all pranizae collected in light traps would be 2–120 h post-feeding. Because there is no control over the amount of time passed from gnathiid-host detachment to the gnathiid entering the light trap, we analyzed the feasibility of host identification from gnathiid blood meals which had been digested for up to 120 h.

The DNA extraction method described consistently produced extractable DNA of sufficient quality and quantity to accommodate 2–3 PCR reactions, based on using 50 ng of DNA template (Hendrick, unpublished data). While template concentration needed to be doubled or reamplified with select samples, the 50 ng DNA template concentration resulted in good overall host identification for the majority of the samples analyzed. Thus, the fish *cox*1 primers used are quite sensitive considering the finite quantity of fish host DNA present within the total extracted DNA composed primarily of gnathiid source. These findings validate the described method delivering sufficient quantity and quality of *cox*1 amplicon for reproducible and accurate fish host identification in future investigations focused on blood meal sourced from wild-caught gnathiids.

DNA sequencing of gnathiid blood meals has previously been used for host identification of two other gnathiid species from the Great Barrier Reef [43, 44]. However, as field parameters (e.g. temperature) and host diversity may impact performance of our molecular-based host species identification method, we conducted a small validation study with wild-caught samples. While only 69% of samples (*n *= 100) resulted in a ≥ 98 % match to a fish host, our method resulted in significantly better sensitivity than previous reported methods resulting in a host species match ≥ 98% for only 21.7% of samples [44]. It is important to note that the successful host identification in both studies is dependent on the availability of reference sequences in public databases.

The lower fish host identification success rate (69.0%) we observed in comparison to the controlled feeding study with known host fish is likely due to a combination of factors. Blood meal volume could be affected by the ease to which gnathiids attach to specific hosts. In particular, when gnathiids feed in the wild, environmental factors or behaviors of the host may influence the volume of the blood meal obtained and possibly lower the amount of DNA template recovered for molecular analyses. In addition, host behaviors, including rubbing against objects, could cause the gnathiid to detach prematurely and thus reduce total blood meal volume intake. These behaviors have been reported for multiple species of fishes in response to ectoparasite exposure [45–47]. The > 3-fold lower average concentration of DNA recovered from wild-fed gnathiids (10.8 ± 4.7 ng/µl; *n *= 37) compared to extracted DNA from gnathiids collected at 0800 h during our controlled experiments (37.3 ± 30.2 ng/µl; *n *= 19) corresponds to these previous studies.

The physical conditions within lighted plankton traps could also play a role in the degradation of host DNA during field collection. It is important to note that during our controlled experimental feeding study, only blood meals from gnathiids that were alive (gnathiids were seen swimming in a Petri dish under a stereomicroscope) at the time of preservation were analyzed. Gnathiid mortality prior to preservation may contribute to an accelerated DNA degradation rate. Light traps attract multiple types of zooplankton and trap “loads” can often be heavy. This in turn can lead to rapid oxygen depletion within the trap, and ultimately death of the contents by asphyxiation. To mitigate this, traps can be fitted with “ventilation” holes at the end opposite the light and/or by the addition of oxygen tablets. Upon collection of the light traps, the contents can be emptied into an aerated container, during transport and while awaiting processing, to further address problems associated with high plankton loads.

In the controlled feeding study, a barred hamlet (*Hypoplectrus puella*), a highly susceptible host of *Gnathia marleyi*, was used to validate the host sequence identification in this study. However, gnathiids infect a broad range of fish hosts with some species being more susceptible or heavily exploited than others [17, 40, 44]. While our field validation study, along with existing observational data, suggest that fish are the primary host of this marine ectoparasite, we have observed gnathiid isopods attaching to invertebrates collected in lighted plankton traps, such as chaetognaths and small polychaetes. Interestingly, 25% of blood meal sequences from wild-caught gnathiids showed probable alignment to invertebrate species, such as those listed above, although matches to sequences in BLAST were much lower than 98%. While feeding on invertebrate hosts could be due to the artificial environment caused by trapping gnathiids and other invertebrates within a light trap, further studies are needed.

In a previous study identifying fish hosts of gnathiids on the Great Barrier Reef, *12S* rDNA primers were found to loosely match to invertebrate species within the order Diptera. However, it was stated that these sequences could potentially be from the gnathiids themselves [43]. To test the possibility of amplifying gnathiid DNA using our *cox*1 primers, specifically designed to amplify fish DNA, DNA was purified from unfed juvenile gnathiids (zuphea). Although the sample group is small, three samples from newly metamorphosed third-stage zuphea exclusively amplified the known fish host DNA, likely due to an incomplete metamorphosis. No other zuphea samples tested (*n* = 18) resulted in amplification of host fish or invertebrate DNA, validating no primer cross reactivity with gnathiid DNA. Ongoing efforts to develop new invertebrate specific *cox*1 primers will enable more definitive invertebrate host species identification.

A small subset of the analyzed wild-fed gnathiids (6 of 100), did not result in a BLAST match that may be due to absence of other fish *cox*1 sequences from this public database. Alternatively, insufficient quantity and/or quality of host DNA was recovered to generate a detectable sequence or readable chromatogram.

## Conclusions

To our knowledge, this is the first study to methodically examine, under controlled conditions, the integrity of host DNA over time within gnathiid isopod blood meals, in efforts to deliver an accurate, reproducible, and robust molecular diagnostic tool for fish host identification. Further testing in a small field study supports the use of our molecular-based procedure for future field projects. A very interesting finding from this preliminary field study is that *G. marleyi* also seems to exploit non-fish hosts, which supports observational data from our team. Therefore, future sampling efforts and analyses will include developing additional diagnostic primer sets in efforts to correctly identify the diversity of gnathiid isopod hosts. To determine the breadth of hosts used by various species of gnathiids in coral reef environments, where host diversity is high, this systematic degradation study of gnathiid blood meals establishes important metrics to inform sample collection and handling techniques. By determining the full range of gnathiid isopod hosts, we can gain insight into host-parasite interactions that contribute to the overall health of host populations and coral reef communities.

## Additional file

**Additional file 1: Table S1.** List of host species identified using blood meals from wild-fed gnathiids. The list includes both common names and scientific names for identified hosts of *Gnathia marleyi*, as well as the accession numbers of GenBank reference sequences.

## Data Availability

Data supporting the conclusions of this article are included within the article and its additional file. The sequences generated in the present study were submitted to the GenBank database under the accession numbers MN046177-MN046204.
