# Supplementary material for: Host DNA integrity within blood meals of hematophagous larval gnathiid isopods (Crustacea, Isopoda, Gnathiidae)
Source: Parasit Vectors. 2019 Jun 24;12:316. doi: 10.1186/s13071-019-3567-8 (PMC6591976; doi:10.1186/s13071-019-3567-8)
Supplement: Supplementary file 1 — Additional file 1: Table S1. List of host species identified using blood meals from wild-fed gnathiids. The list includes both common names and scientific names for identified hosts of Gnathia marleyi, as well as the accession numbers of GenBank reference sequences. [file 13071_2019_3567_MOESM1_ESM.pdf]

Additional file 1: Table S1. List of host species identified using blood meals from wild-fed gnathiids. The list includes both common names and scientific names for identified hosts of *Gnathia marleyi* , as well as the accession numbers of GenBank or BOLD reference sequences.

**Family**	**Species**	**Common Name**	**Reference Sequence Accession Number**	**Number of Samples Matching Reference**	**GenBank Accession Numbers for Consensus Sequences Generated in this Study**
Acanthuridae	*Acanthurus bahianus*	Ocean Surgeon	JQ839700.1	2	
	Acanthurus coeruleus	Blue tang	KC623659.1	2	
Carangidae	*Carangoides ruber*	Bar Jack	MH777710.1	5	MN046186, MN046199, MN046204
	Trachinotus falcatus	Permit	BOLD:AAB9494	2	MN046198, MN046202
Gerreidae	*Gerres cinereus*	Yellow Fin Mojarra	KT005474.1	6	MN046177, MN046178, MN046181
Haemulidae	*Haemulon carbonarium*	Caesar Grunt	JQ841204.1	1	MN046183
	Haemulon flavolineatum	French Grunt	JQ842143.1	5	MN046192, MN046200
	Haemulon macrostomum	Spanish Grunt	GU225307.1	1	MN046190
	Haemulon parra	Sailor's choice	JQ841908.1	1	
	Haemulon plumierii	White Grunt	KY402342.1	6	MN046182
	Haemulon sciurus	Bluestripped Grunt	JQ840088.1	3	MN046185, MN046193
Holocentridae	*Holocentrus adscensionis*	Squirrelfish	KX961690.1	1	
	Holocentrus rufus	Longspine Squirrelfish	JQ840538.1	3	MN046184, MN046191, MN046197
Labridae	*Halichoeres bivittatus*	Slippery Dick	JQ842883.1	1	
Lutjanidae	*Lutjanus analis*	Mutton Snapper	JX297373.1	2	MN046194
	Lutjanus apodus	Schoolmaster Snapper	JQ842557.1	3	MN046188
	Lutjanus purpureus	Red snapper	JX297373.1	1	
	Lutjanus synagris	Lane Snapper	JQ841932.1	1	
	Ocyurus chrysurus	Yellow Tail Snapper	KX119476.1	8	MN046179, MN046180, MN046201, MN046203
Muraenidae	*Gymnothorax funebris*	Green Moray	MF041569.1	1	
Pomacentridae	*Abudefduf saxatilis*	Sergeant Major	MF988091.1	1	
	Stegastes adustus	Dusky Damsel	JQ839904.1	1	
Sparidae	*Calamus bajonado*	Jolthead Porgy	KJ012301.1	1	
	Calamus penna	Sheepshead Porgy	JQ842798.1	4	MN046189, MN046196

Calamus pennatula	Pluma Porgy	BOLD:AAF8746	1	MN046187
Calamus proridens	Littlehead Porgy	KJ012315.1	6	

Accessed on GenBank: 10 August 2018
